# Integrated transcriptomic and metabolomic analysis reveals developmental stage-specific molecular responses to phosphorus deficiency in soybean

**DOI:** 10.3389/fpls.2025.1692661

**Published:** 2025-10-08

**Authors:** Xiulin Liu, Xueyang Wang, Chunlei Zhang, Fengyi Zhang, Kezhen Zhao, Rongqiang Yuan, Sobhi F. Lamlom, Bixian Zhang, Honglei Ren

**Affiliations:** ^1^ Soybean Research Institute of Heilongjiang Academy of Agriculture Sciences, Harbin, China; ^2^ Plant Production Department, Faculty of Agriculture Saba Basha, Alexandria University, Alexandria, Egypt; ^3^ Institute of Biotechnology of Heilongjiang Academy of Agricultural Sciences, Harbin, China

**Keywords:** phosphorus deficiency, multi-omics integration, transcription factors, gene-metabolite networks, plant adaptation mechanisms, abiotic stress response

## Abstract

**Background and knowledge gap:**

Phosphorus (P) deficiency is a major constraint to crop productivity worldwide, yet the molecular mechanisms behind stage-specific responses to severe P limitation during soybean development are not well understood. Although previous studies have looked at P stress responses, comprehensive multi-omics analyses across different developmental stages are missing, which limits our understanding of how P-efficient cultivars manage metabolic and transcriptional responses throughout their growth cycle.

**Objectives and methods:**

This study used an integrated transcriptomic and metabolomic approach to analyze stage-specific responses to severe phosphorus limitation (99.875% reduction) in the P-efficient soybean cultivar Heinong 551 across four developmental stages: trefoil, flowering, podding, and post-podding.

**Results:**

Metabolomic profiling identified 280 differentially expressed metabolites (DEMs) during trefoil and 851 during flowering, showing a threefold increase in metabolic disturbance during reproductive development. Transcriptomic analysis revealed 15,401 differentially expressed genes (DEGs) across stages, with 94% occurring in early phases (trefoil: 3,825; flowering: 10,660). Functional enrichment showed stage-specific responses, with the trefoil stage enriched in cell wall and membrane processes, and flowering enriched in photosynthesis, isoflavonoid biosynthesis, and cuticle development. Transcription factor analysis identified 87 differentially expressed transcription factors from 31 families, mainly *bHLH*, *bZIP*, and *WRKY*. Integrated multi-omics analysis under strict criteria (correlation coefficient |r| > 0.9) revealed networks between transcripts and metabolites, with flowering showing increased transcriptional control over metabolism. Key trade-offs included a shift from sucrose export to starch storage, suppression of nitrogen enzymes, and activation of antioxidant defenses despite oxidative damage. Physiological principal component analysis explained 92% of variance, distinguishing treatment groups and three metabolic clusters: carbon assimilation/export, nitrogen assimilation, and stress response.

**Conclusion:**

Carbon metabolism exhibited compensatory mechanisms, including increased RubisCO and invertase activities, while nitrogen metabolism involved the downregulation of nitrate reductase, glutamine synthetase, and protein content. These findings reveal stage-specific molecular strategies used by P-efficient soybeans under severe limitation and inform sustainable agriculture practices aimed at optimizing crop performance in phosphorus-deficient conditions.

## 1 Introduction

Soybean (*Glycine max* L. Merr.) represents one of the world’s most economically important legume crops, providing essential protein and oil for both human consumption and animal feed (Lamlom et al., 2020; Siddique et al., 2024; Kumari et al., 2025). As a nitrogen-fixing legume, soybean has particularly high phosphorus requirements to support both nodulation processes and seed development (Gaguan and Nagal, 2025). The crop’s sensitivity to phosphorus limitation makes it an ideal model system for understanding plant adaptation mechanisms to nutrient stress (Shahid et al., 2009; Balemi and Negisho, 2012; Liu et al., 2024). Current agricultural practices rely heavily on phosphorus fertilizers derived from finite rock phosphate reserves, making the development of phosphorus-efficient crop varieties increasingly critical for sustainable agriculture (Vance et al., 2003; Cordell and White, 2013).

Plant responses to phosphorus deficiency involve complex, multi-layered regulatory networks that coordinate metabolic, physiological, and developmental adjustments (Khan et al., 2023; Luo et al., 2023). At the molecular level, phosphorus stress triggers extensive transcriptional reprogramming involving numerous transcription factor families, including *MYB, WRKY, bHLH*, and *NAC*, which orchestrate the expression of genes involved in phosphorus acquisition, mobilization, and recycling (Chu et al., 2020; Prodhan et al., 2022; Zhou et al., 2023). Metabolically, plants undergo significant biochemical adjustments, including alterations in carbon partitioning, amino acid metabolism, and secondary metabolite biosynthesis, to optimize resource utilization under phosphorus-limited conditions (Zhang et al., 2014a; Plaxton and Lambers, 2015; Ren et al., 2023). Phosphorus is a crucial macronutrient essential for plant growth, development, and metabolism. Phosphorus is an indispensable element in nucleic acids, phospholipids, and energy-transferring molecules such as ATP, rendering it crucial for fundamental biological processes, including photosynthesis, respiration, and protein synthesis (Vance et al., 2003; Lambers et al., 2015; Roychowdhury et al., 2023a). Despite its biological importance, phosphorus availability in agricultural soils is often limited due to its tendency to form insoluble complexes with soil minerals, particularly in alkaline and acidic conditions (Raghothama, 1999; Thomas Sims and Pierzynski, 2005; De Bang et al., 2021). This limitation poses significant challenges for global crop production, as phosphorus deficiency can severely constrain plant growth and reduce agricultural yields (Hernández et al., 2007; George and Richardson, 2008; Ren et al., 2024b).

Recent advances in high-throughput sequencing and mass spectrometry technologies have enabled comprehensive systems-level analyses of plant stress responses (Song et al., 2016; Xue et al., 2018). Multi-omics approaches, particularly the integration of transcriptomics and metabolomics, provide unprecedented insights into the molecular mechanisms underlying plant adaptation to environmental stress (Roychowdhury et al., 2023b; Haidar et al., 2024; Ren et al., 2024a; Satrio et al., 2024; Ren et al., 2025). These integrated analyses reveal the intricate relationships between gene expression changes and metabolic reprogramming, offering a holistic understanding of how plants coordinate their responses to nutrient limitation across different developmental stages (Satrio et al., 2024; Wang et al., 2024; Sharma et al., 2025). The temporal dynamics of plant stress responses add another layer of complexity to phosphorus deficiency adaptation (Jain et al., 2007; Zhang et al., 2014b). Different developmental stages exhibit varying sensitivities to nutrient stress, with reproductive stages often showing heightened vulnerability due to increased metabolic demands for flower and seed development (Khan et al., 2023; Zhang et al., 2023; Zha et al., 2025; Zhang et al., 2025a, 2025). Understanding these stage-specific responses is crucial for developing targeted breeding strategies and agricultural management practices that optimize crop performance under phosphorus-limiting conditions (Skalak et al., 2021; Mishra et al., 2024).

While previous studies have explored how plant species respond to phosphorus stress, comprehensive multi-omics analyses across soybean development stages are rare. Most research has focused separately on transcriptomic or metabolomic responses, leaving gaps in understanding the molecular networks that control adaptation. This study aims to fill these gaps by combining transcriptomic and metabolomic analyses to investigate soybean responses at four key stages: trefoil, flowering, podding, and post-podding. Using the phosphorus-efficient cultivar Heinong 551, we studied the mechanisms that enable adaptation under a 99.875% phosphorus reduction. Our objectives were to: (1) characterize stage-specific transcriptomic and metabolomic responses to phosphorus deficiency, (2) identify key transcription factors and pathways involved, and (3) reveal the trade-offs and strategies enabling soybean to withstand severe phosphorus shortages. The findings illuminate the molecular basis of phosphorus stress tolerance in soybean and identify potential targets for breeding more phosphorus-efficient crops. By elucidating the networks of genes, metabolites, and regulatory processes, this research deepens our understanding of plant nutrient stress adaptation and supports sustainable farming practices aimed at boosting crop productivity amid nutrient limitations.

## 2 Materials and methods

### 2.1 Plant material and experimental design

This study utilized soybean cultivar Heinong 551 from the Soybean Research Institute of Heilongjiang Academy of Agricultural Sciences, selected for its tolerance to phosphorus-limited conditions and superior performance under nutrient stress, making it ideal for studying molecular adaptation mechanisms. The experiment employed a randomized design with eight pots per treatment group (control and low phosphorus), with two seeds per pot initially, later thinned to one plant. Plants were grown in washed vermiculite under controlled environmental conditions: 28 °C day/22 °C night temperatures, 12-hour light/dark photoperiod, and 80% relative humidity, with continuous monitoring to prevent environmental fluctuations. The phosphorus stress system followed established protocols to ensure reproducibility. For the control group (adequate phosphorus), 10 g of insoluble Ca_10_(PO_4_)_6_(OH)_2_ was mixed with 15 kg of vermiculite to provide baseline phosphorus availability, and plants received complete 1× Hoagland solution containing 2000 μmol·L^−^¹ KH_2_PO_4_. For the low phosphorus treatment, no additional phosphorus was added to the vermiculite medium, and plants received modified 1× Hoagland solution with only 2.5 μmol·L^−^¹ KH_2_PO_4_, representing a 99.875% reduction compared to standard levels. To prevent potassium deficiency, KCl (1997.5 μmol·L^−^¹)was added to maintain adequate potassium concentrations in the low phosphorus solution. Treatment application began at the emergence of the third trifoliate leaf, a developmental stage characterized by established root systems capable of responding to stress and exhibiting transcriptional plasticity. The 14-day treatment duration was selected to allow sufficient time for molecular responses to develop while avoiding severe plant damage that could confound results.

### 2.2 Sample collection and preparation

Samples for Heinong 551 analysis were collected at four key developmental stages under control and phosphorus-deficient conditions to capture molecular adaptations. The trefoil stage (21 days post-germination) (samples A and B) represented early vegetative growth, the flowering stage (45 days) (C and D) marked the transition to reproductive development, the podding stage(65 days) (E and F) indicated initial reproductive investment, and the post-podding stage (85 days) (G and H) reflected seed maturation. Healthy young roots were harvested at the three-leaf stage in August 2024 for root analysis. For developmental stage analysis, 0.1 g leaf samples from each stage were collected, flash-frozen in liquid nitrogen to preserve molecular integrity.

### 2.3 Integrated sampling strategy for multi-omics analysis

The sampling strategy was designed to capture different aspects of molecular adaptation through coordinated transcriptomic and metabolomic analyses at optimal timepoints. For transcriptomic analysis, functional leaves were collected from four randomly selected pots per treatment group after 7 days of phosphorus stress treatment. This early timepoint was chosen to capture immediate molecular responses before visible stress symptoms appeared, focusing on actively photosynthetic and metabolically active leaf tissues. Three biological replicates were established for transcriptomic analysis, with each biological replicate representing independent plants to ensure statistical validity and biological relevance of gene expression changes. Metabolomic analysis was conducted using samples collected after 14 days of treatment, representing a later timepoint that captured established metabolic adaptations to phosphorus stress. Functional leaves were again selected as the target tissue to maintain consistency with transcriptomic analysis and enable direct comparison between gene expression and metabolite changes. Four pots were randomly selected from the remaining treatment replicates for metabolomic analysis, with four biological replicates established to provide robust quantification of metabolite changes. Each biological replicate underwent four technical measurements to ensure analytical precision and reliability of metabolite quantification data. Sample processing followed strict protocols to preserve molecular integrity and prevent degradation of artifacts. All tissue samples were harvested and immediately flash-frozen in liquid nitrogen within 30 seconds of collection to halt enzymatic activities and preserve RNA and metabolite stability. Frozen samples were subsequently stored at -80°C until molecular analysis, with comprehensive labeling and documentation systems maintaining complete chain of custody throughout the experimental process. This comprehensive sampling approach resulted in a total of 24 samples for developmental analysis and additional samples for comparative analysis, with the remaining eight pots from each treatment group reserved for physiological measurements.

### 2.4 Physiological and biochemical analyses

Comprehensive physiological characterization involved measuring key metabolic parameters related to carbon metabolism, nitrogen assimilation, and antioxidant defense systems. Sucrose and starch contents were quantified using enzymatic assays, while RubisCO activity was assessed spectrophotometrically. Enzymes involved in sucrose metabolism, such as sucrose synthase and invertase, were analyzed using standard protocols. For nitrogen metabolism, total soluble protein levels were determined via Bradford assay, and key enzymes like nitrate reductase, glutamine synthetase, and GOGAT were measured using established enzymatic methods. The antioxidant system was evaluated by measuring malondialdehyde (MDA) levels as a lipid peroxidation marker, along with activities of key antioxidant enzymes including catalase, peroxidase, and superoxide dismutase. All enzyme activities were conducted under standardized conditions with appropriate controls and expressed relative to tissue weight or protein content.

### 2.5 Transcriptomic analysis

Total RNA was extracted using TRIzol (Invitrogen) with soybean-optimized modifications. Frozen tissue was rapidly ground in liquid nitrogen to prevent degradation. The procedure involved purification steps to remove contaminants, phase separation with chloroform, RNA precipitation with isopropanol, and ethanol washes. RNA quality was evaluated via gel electrophoresis and Bioanalyzer, with only samples having RIN > 7.0 used for sequencing. RNA-seq libraries were prepared with the NEBNext Ultra™ RNA Library Prep Kit, starting with mRNA enrichment, fragmentation, and cDNA synthesis. Adapter ligation permitted multiplexing. The sequencing was performed on the Illumina NovaSeq 6000 platform with paired-end reads, followed by quality filtering. Filtered reads were mapped to the soybean genome(*Glycine max*, version Wm82.a2.v1) obtained from the NCBI database (https://www.ncbi.nlm.nih.gov/datasets/genome/GCF 004193775.1)) using STAR. Data were deposited in NCBI SRA (PRJNA1232401). Average read depth of 45 million paired-end reads per sample, withpaired endates >85% and RNA integrity numbers (RIN) >7.0. Expression levels were quantified with RSEM, and differentially expressed genes (DEGs) were identified using edgeR with FDR < 0.05 and p < 0.05. Gene Ontology (GO) and Kyoto Encyclopedia of Genes and Genomes (KEGG) pathway enrichment analyses of DEGs were performed using clusterProfiler v4.8.2 with the Benjamini–Hochberg correction. Pathways with adjusted *p*-values < 0.05 were considered significantly enriched. The fold enrichment was calculated as the ratio of gene frequency in the test set (GenRatio) to gene frequency in the reference set (BgRatio). Differential transcription factor analysis was conducted using the STAMP software, with FDR < 0.05 and *p*-value < 0.05 as the threshold for identifying differentially expressed transcription factors.

### 2.6 Metabolomic analysis

Metabolomic analysis used 100 mg of frozen tissue per sample, weighed precisely for consistency. The extraction employed a methanol-chloroform system to recover polar and non-polar metabolites. Samples were transferred to 2-mL tubes and extracted with 0.3 mL methanol and 0.1 mL chloroform, capturing diverse metabolites. An internal standard of 60 μL ribitol was added for quantification and to correct for variances. Samples were homogenized at 70 Hz for 5 minutes, then incubated at 70 °C for 10 minutes, and centrifuged at 12,000 × g for 10 minutes at 4 °C to separate metabolites. LC-MS analysis followed derivatization for non-volatile metabolites. 0.35 mL supernatant was vacuum dried at 30 °C for 2 hours. Derivatization involved treatment with 80 μL methoxyamine hydrochloride for 2 hours at 37 °C, followed by 100 μL N,O-bis(trimethylsilyl)-trifluoroacetamide with 1% trimethylchlorosilane at 70 °C for 1 hour. Samples were analyzed on an Agilent 7890 GC coupled with a Pegasus HT TOF-MS, with four biological replicates per condition. Data were processed with LECO ChromaTOF software for peak detection and identification using the LECO-Fiehn Rtx5 database. Metabolite data were normalized for sample weight and drift, then analyzed with SIMCA-P 13.0 for PCA, OPLS-DA, and PLS-DA. Metabolites were identified based on t-test (p < 0.05), VIP > 1, and mass spectral similarity scores >700 on a 0–1000 scale using the LECO-Fiehn Rtx5 database. KEGG pathway analysis and MetaboAnalyst 6.0 (www.metaboanalyst.ca) facilitated pathway identification and visualization.

### 2.7 Integrated multi-omics analysis

Integrated analysis of transcriptomic and metabolomic data identified changes in gene expression and metabolite levels, revealing regulatory relationships. Pearson’s correlation coefficients were calculated between differentially expressed genes and metabolites using MetaboAnalyst 6.0 (www.metaboanalyst.ca) for data integration and visualization. Significant associations (|correlation coefficient| > 0.9, P < 0.001) indicated potential regulatory links, pointing to functional connections in metabolic pathways. Cytoscape (version 3.8.2) was used to visualize gene-metabolite interactions and identify key regulatory nodes, such as hub genes and metabolites, involved in metabolic responses to phosphorus stress.

### 2.8 Statistical analysis

Statistical analysis used a hierarchical approach suitable for the data, with one-way ANOVA for physiological measurements to detect differences among groups, followed by Tukey’s HSD *post-hoc* tests. Student’s t-test evaluated treatment differences at P < 0.05, with results as mean ± SD. Spearman’s correlation assessed relationships without assuming normality. Data quality checks included controls, blanks, and replicates, with assumption tests for normality, homogeneity, and independence. Violations prompted non-parametric methods or transformations for valid analysis.

## 3 Results

### 3.1 Metabolite profiling reveals distinct stage-specific metabolic signatures under phosphorus deficiency

**Figure 1:**
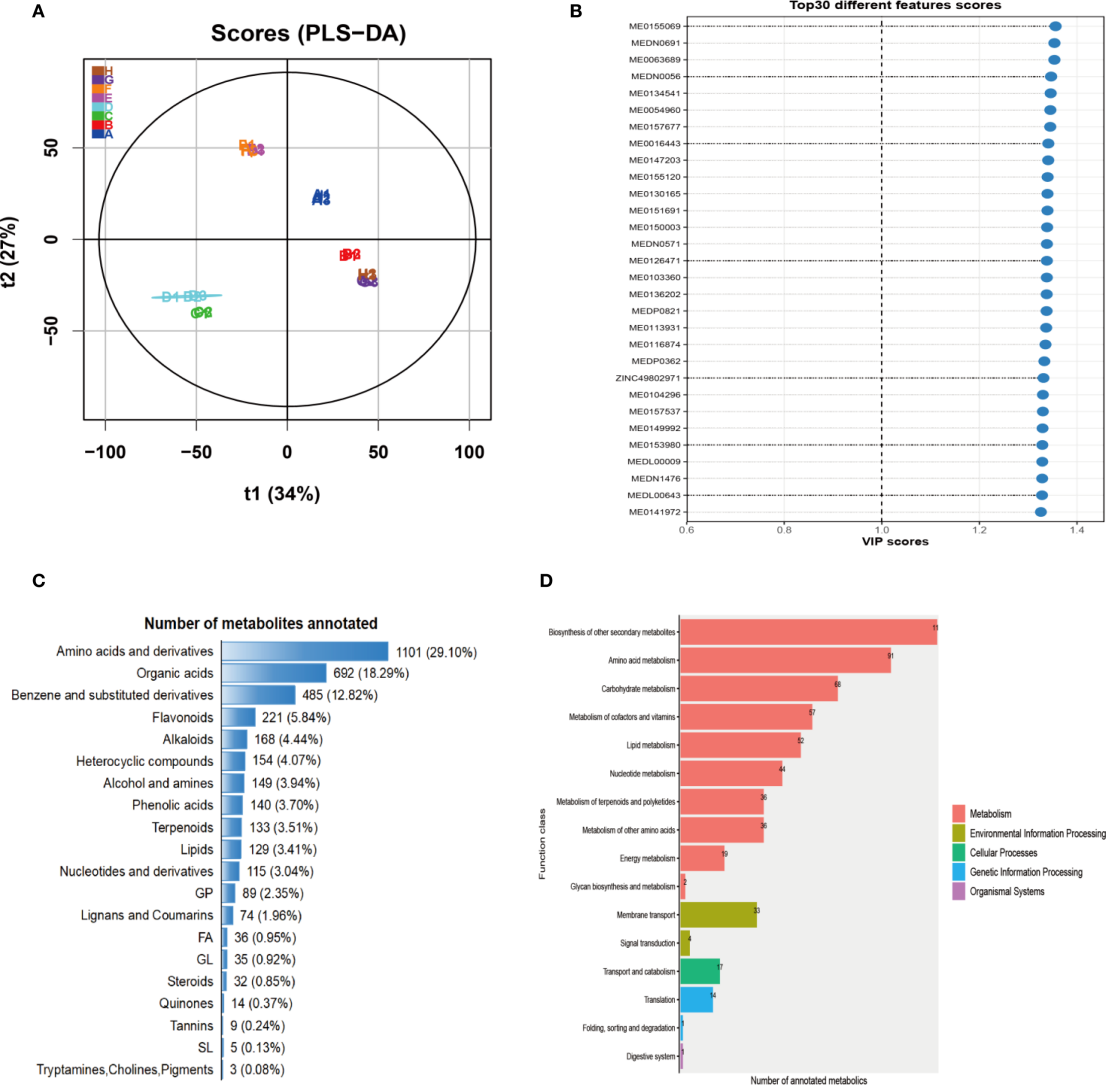
Statistical analysis and annotation of metabolites. **(A)** PLS-DA score plot for each developmental period based on LC-MS analysis. **(B)** PLS-DA VIP scores for individual metabolites, showing the top 30 metabolites with the highest variable importance in projection values. **(C)** Taxonomic classification of metabolites, with amino acids and derivatives accounting for the largest proportion. **(D)** Statistical annotation of metabolites using the KEGG database.

To characterize global metabolic alterations under phosphorus (P) stress, we performed LC-MS-based metabolomic profiling across different developmental stages. Partial least squares discriminant analysis (PLS-DA) revealed a clear separation between the trefoil and flowering stages, as well as between the control and treatment groups (**Figure 1A**). The first two components accounted for 61% of the total variance (t1: 34%, t2: 27%), indicating a strong stage-dependent metabolic divergence driven by P availability. Variable Importance in Projection (VIP) analysis identified the top 30 metabolites that contributed most significantly to group discrimination (**Figure 1B**). These key metabolites exhibited VIP scores greater than 1, suggesting their robust involvement in phosphorus-responsive metabolic pathways. Many of these metabolites were differentially accumulated in the flowering stage, consistent with the higher metabolic demand and perturbation observed during reproductive development. Metabolite taxonomy classification showed that amino acids and their derivatives represented the most abundant chemical class (29.1%), followed by organic acids (18.3%), benzene and substituted derivatives (12.8%), and flavonoids (5.8%) (**Figure 1C**). This profile reflects both primary metabolic adjustments and secondary metabolite responses associated with stress signaling and defense. Functional annotation using the KEGG database mapped annotated metabolites into diverse biological pathways, with enrichment primarily in metabolism-related categories (**Figure 1D**). The most enriched pathways included biosynthesis of secondary metabolites, amino acid metabolism, carbohydrate metabolism, and vitamin and lipid metabolism. Additionally, a subset of metabolites was linked to cellular processes (e.g., membrane transport), environmental information processing (e.g., signal transduction), and genetic information pathways (e.g., transcription and protein folding), indicating a broad impact of P deficiency on cellular function and regulatory networks. Together, these results demonstrate that P deficiency induces widespread and developmentally distinct metabolic reprogramming, with amino acid turnover, organic acid flux, and secondary metabolite biosynthesis emerging as central adaptive strategies. The identified metabolite classes and enriched pathways provide candidate targets for improving phosphorus use efficiency and stress resilience in crops.

### 3.2 Phosphorus deficiency triggers developmentally distinct metabolic reprogramming

**Figure 2:**
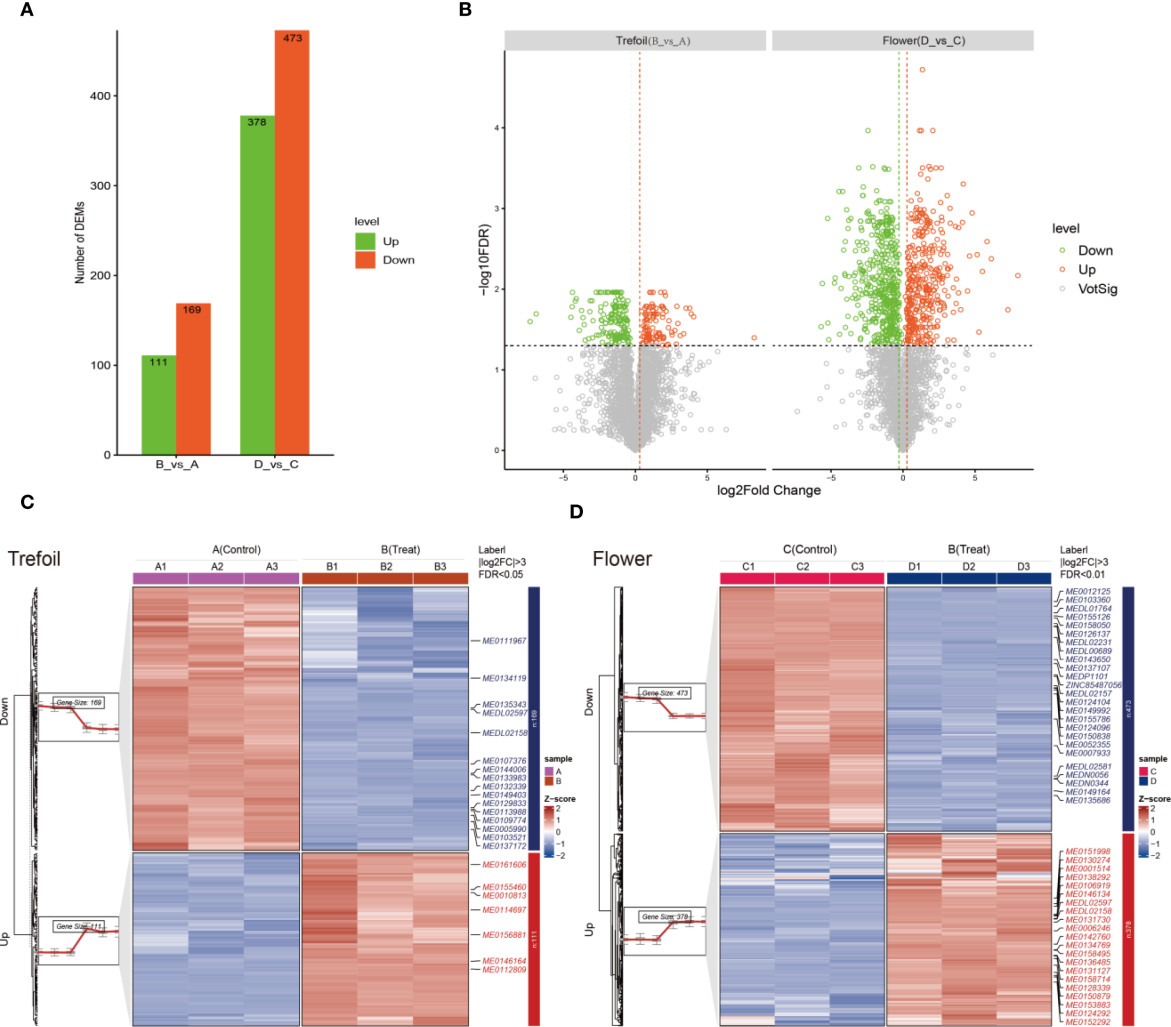
Metabolite analysis of low-phosphorus stress at two developmental time points. **(A)** Comparison between treatment and control groups across both developmental periods (trefoil: treatment **(B)** vs. control **(A)**; flowering: treatment **(D)** vs. control **(C)**). **(B)** Differential metabolite analysis showing up- and down-regulated metabolites across the two comparison groups. Significantly up-regulated metabolites (VIP > 1, log_2_FC > 1, and P < 0.05) are shown in red, while significantly down-regulated metabolites (VIP > 1, log_2_FC < -1, and P < 0.05) are displayed in green. **(C, D)** Clustering heatmaps displaying the expression patterns of differentially expressed metabolites (DEMs). In the trefoil period, 111 DEMs were up-regulated and 169 DEMs were down-regulated in treatment **(B)** relative to control **(A)**. In the flowering period, 378 DEMs were up-regulated and 473 DEMs were down-regulated in treatment **(D)** relative to control **(C)**. Sample sizes (n=3 for transcriptomic analysis, n=3 for metabolomic analysis).

To assess the metabolic effects of phosphorus deficiency across different developmental stages, we compared metabolite profiles between treatment and control groups at the trefoil (vegetative) and flowering (reproductive) stages. We identified a total of 280 DEMs during the trefoil stage (111 up-regulated, 169 down-regulated), while the flowering stage showed a much stronger response, with 851 DEMs (378 up-regulated, 473 down-regulated) (VIP > 1, log_2_FC > 1, P < 0.05) (**Figure 2A**). This approximately threefold increase in affected metabolites during flowering indicates increased metabolic sensitivity to P limitation during reproductive development. Volcano plot analysis demonstrated clear divergence in the fold-change and significance of DEMs between the two stages (**Figure 2B**). In the trefoil stage, most DEMs fell within a ±2 log_2_ fold-change range, indicating moderate metabolic adjustments. Conversely, the flowering stage displayed a wider range of fold changes and higher statistical significance, reflecting more extensive metabolic reprogramming. Up-regulated metabolites (VIP > 1, log_2_FC > 1, P < 0.05) are shown in red, and down-regulated metabolites (VIP > 1, log_2_FC < –1, P < 0.05) in green, revealing stage-specific patterns of metabolic activation and suppression. Hierarchical clustering further supported these findings, showing distinct expression patterns of DEMs between control and P-deficient plants at both stages (**Figures 2C, D**). In the trefoil stage, clustering of 280 DEMs clearly separated control samples (A1–A3) from treated samples (B1–B3), with coordinated downregulation of biosynthetic metabolites and upregulation of stress-related compounds. The flowering stage presented a more complex heatmap with broader metabolic shifts, including 378 metabolites that were consistently up-regulated and 473 metabolites that were consistently down-regulated across biological replicates (C1–C3 vs. D1–D3), reinforcing the idea of increased metabolic remodeling during reproductive development. These results indicate that phosphorus deficiency elicits tightly regulated, developmentally specific metabolic responses. The dominant trend of downregulation in both stages suggests a resource-conservation strategy under nutrient stress, while the increased magnitude and diversity of changes at flowering underscore the vulnerability and metabolic demands of reproductive tissues.

### 3.3 Differentially expressed genes across developmental stages

**Figure 3:**
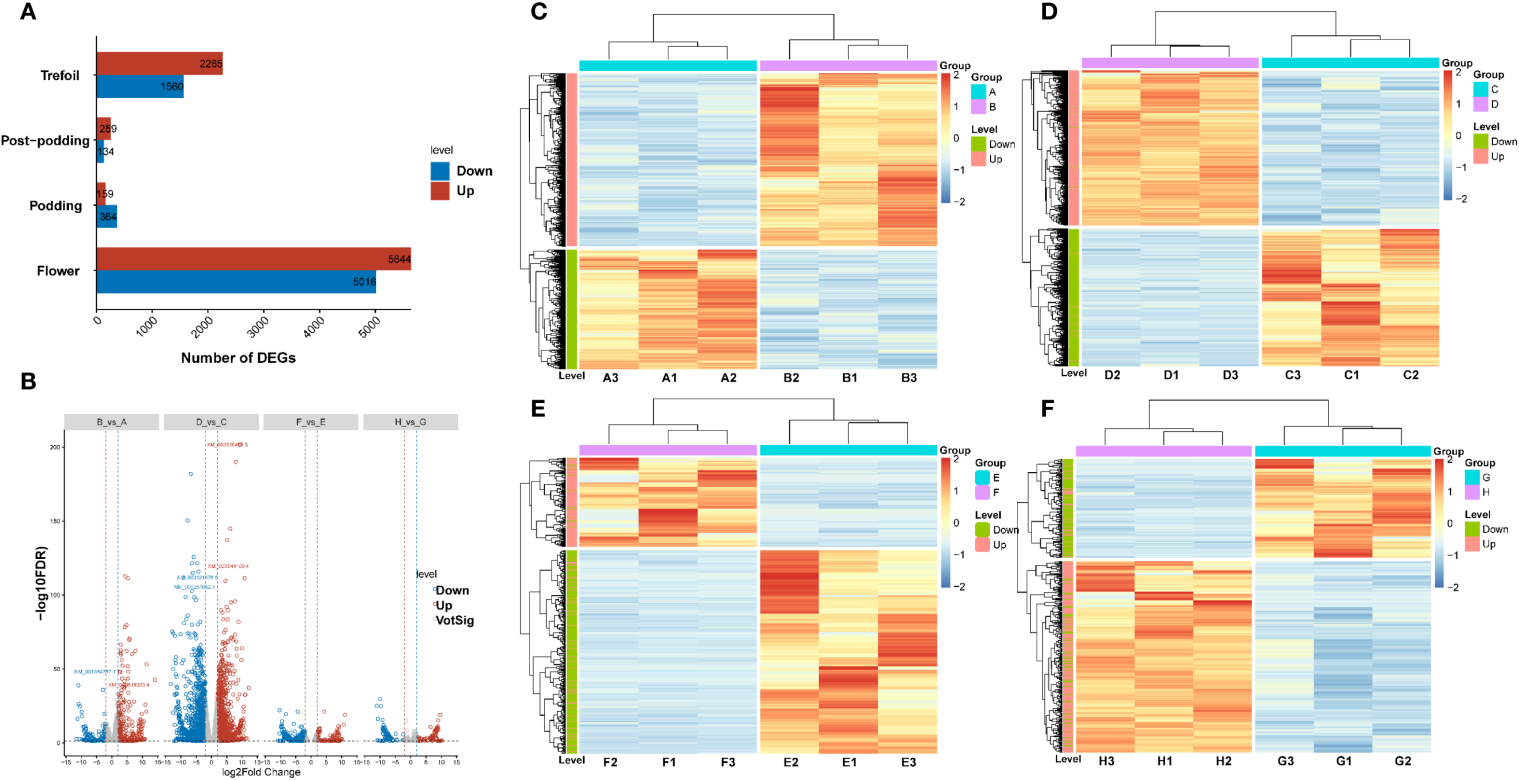
Transcriptomic analysis of low-phosphorus stress responses across soybean developmental stages. **(A)** Bar chart showing the number of differentially expressed genes (DEGs) identified at four developmental stages (trefoil, post-podding, podding, and flower) under low-phosphorus stress conditions. Red bars indicate up-regulated genes and blue bars indicate down-regulated genes. **(B)** Volcano plot displaying the distribution of gene expression changes across all developmental stages. The x-axis represents log_2_ fold change (log_2_FC (-14.67 to 14.11)) and y-axis shows -log_10_ (FDR). Red dots represent significantly up-regulated genes, blue dots represent significantly down-regulated genes, and gray dots represent non-significantly changed genes. Vertical dashed lines indicate fold change thresholds. **(C-F)** Hierarchical clustering heatmaps showing temporal expression patterns of DEGs across sample groups: **(C)** Group A samples (A3, A1, A2, B2, B1, B3), **(D)** Group D samples (D2, D1, D3, C3, C1, C2), **(E)** Group F samples (F2, F1, F3, E2, E1, E3), and **(F)** Group H samples (H2, H1, H2, G3, G1, G2). Color scale represents normalized expression levels from low (blue) to high (red). Dendrograms show hierarchical clustering of both genes (rows) and samples (columns). Side color bars indicate treatment groups and developmental stages.

The analysis of differential expression revealed differing numbers of DEGs between treatment and control conditions at the four developmental stages (**Figure 3A**). We identified 3,825 DEGs in the trefoil stage (B vs A) with 2,265 up-regulated and 1,560 down-regulated, 10,660 DEGs in the flower stage (D vs C) with 5,644 up-regulated and 5,016 down-regulated, 523 DEGs in the podding stage (F vs E) with 159 up-regulated and 364 down-regulated, and 393 DEGs in the post-podding stage (H vs G), consisting of 259 up-regulated and 134 down-regulated. Out of a total of 15,401 DEGs identified across all stages, 25% were found in the trefoil stage, 69% in the flower stage, and 3% each in the podding and post-podding stages, which suggests that low-phosphorus treatment mainly impacts early developmental stages. Further scrutiny of significant DEGs, using rigorous filtering criteria (|log2FC|≥11 & FDR<0.01), revealed 39 highly differential genes (**Figure 3B**). The log2FC values spanned from -11.57 to 14.11 in the trefoil stage, -14.67 to 12.17 in the Flower stage, -10.90 to 11.06 in the podding stage, and from -11.11 to 10.31 in the post-podding stage (**Figure 3B**). K-means clustering of DEGs at each developmental stage displayed two distinct expression patterns for both treatment and control groups (**Figures 3C-F**), verifying the differential response to low-phosphorus stress throughout the developmental stages.

### 3.4 Functional enrichment analysis of DEGs

**Figure 4:**
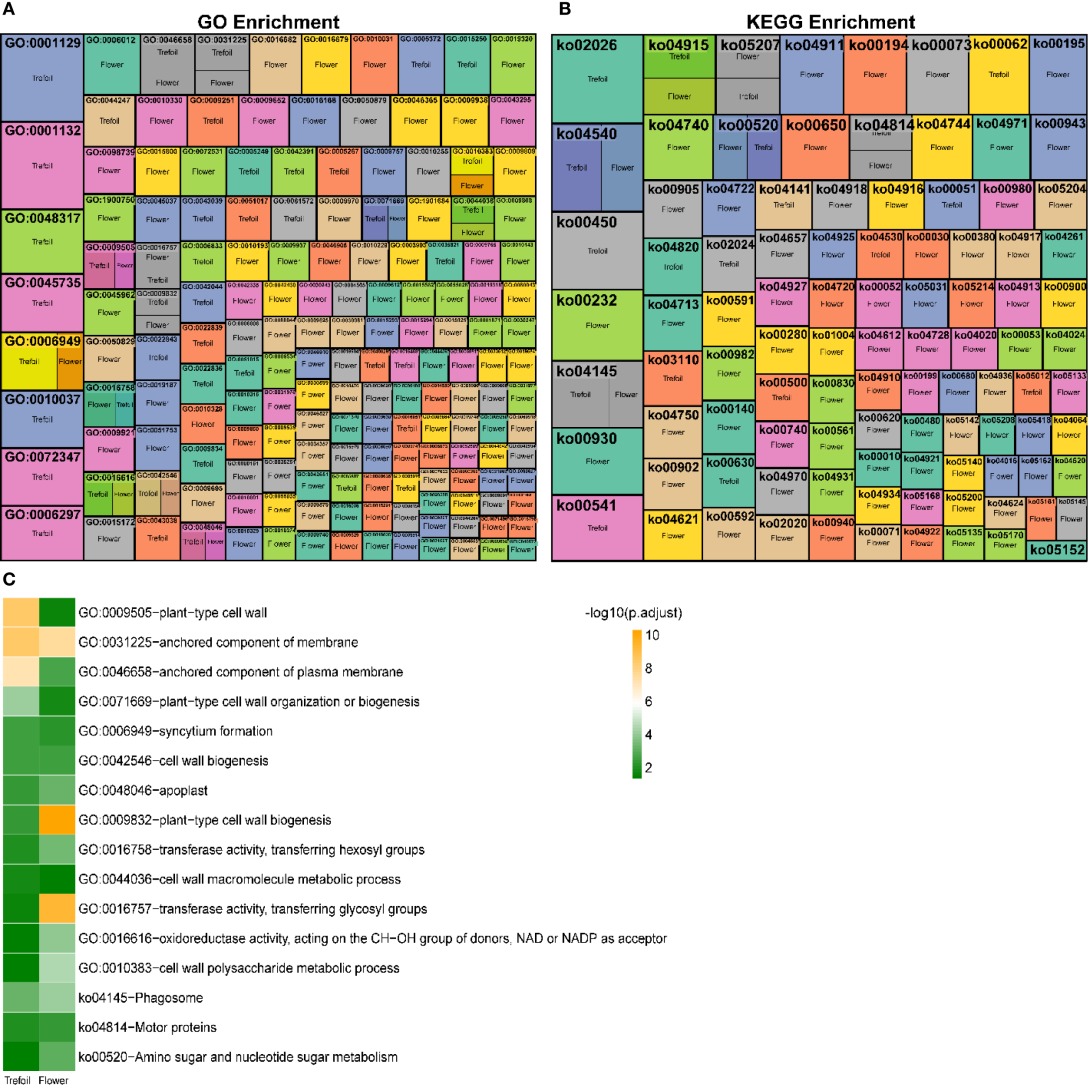
Functional enrichment analysis of differentially expressed genes under low-phosphorus stress. **(A)** Gene Ontology (GO) enrichment analysis heatmap showing significantly enriched biological processes across trefoil and flower developmental stages. Each cell represents the -log_10_(p-value) of enrichment significance, with color intensity indicating the degree of significance. **(B)** KEGG pathway enrichment analysis heatmap displaying significantly enriched metabolic and signaling pathways. Color scale represents enrichment significance levels. **(C)** Summary bar chart of the most significantly enriched GO terms and KEGG pathways, ranked by -log_10_(p.adjust) values. Green bars indicate trefoil stage and orange bars indicate flower stage enrichment.

Since 94% of DEGs were concentrated in the trefoil and flower stages, we focused our functional characterization on these early developmental periods. Gene Ontology (GO) enrichment analysis revealed 44 significantly enriched terms in the trefoil stage, with 21 terms enriched in down-regulated DEGs and 23 terms in up-regulated DEGs (**Figure 4A**). Using stringent criteria (p.adjust<0.001 and log2FoldEnrichment>1.5), we identified four key GO terms: “anchored component of membrane” (GO:0031225), “plant-type cell wall” (GO:0009505), “anchored component of plasma membrane” (GO:0046658), and “plant-type cell wall organization or biogenesis” (GO:0071669). In the flower stage, 137 GO terms were significantly enriched, with 55 terms associated with down-regulated DEGs and 82 with up-regulated DEGs. Nineteen key GO terms met our stringent criteria, including five terms enriched in down-regulated DEGs related to glucosyltransferase activity, response to ozone, and oxidoreductase activity. The remaining 14 terms, enriched in up-regulated DEGs, were predominantly associated with chloroplast thylakoid, plastid thylakoid, thylakoid membrane, photosynthetic membrane, and cuticle development. KEGG pathway analysis identified eight significantly enriched pathways in the trefoil stage (two associated with down-regulated DEGs, six with up-regulated DEGs) and 21 pathways in the flower stage (15 associated with down-regulated DEGs, six with up-regulated DEGs) (**Figure 4B**). Key pathways (p.adjust<0.001 and log2FoldEnrichment>1.5) included “Protein processing in endoplasmic reticulum” (ko04141) and “Phagosome” (ko04145) in the trefoil stage, and “Isoflavonoid biosynthesis” (ko00943), “alpha-Linolenic acid metabolism” (ko00592), “Butanoate metabolism” (ko00650), “Photosynthesis” (ko00195), and “Cutin, suberine and wax biosynthesis” (ko00073) in the flower stage. We identified 13 GO terms and three KEGG pathways that were significantly enriched (p.adjust<0.05) in both trefoil and flower stages (**Figure 4C**). The common GO terms were primarily related to cell wall organization, biogenesis, and metabolism, while the shared KEGG pathways included “Phagosome” (ko04145), “Motor proteins” (ko04814), and “Amino sugar and nucleotide sugar metabolism” (ko00520).

### 3.5 Transcription factor dynamics under low-phosphorus stress

**Figure 5:**
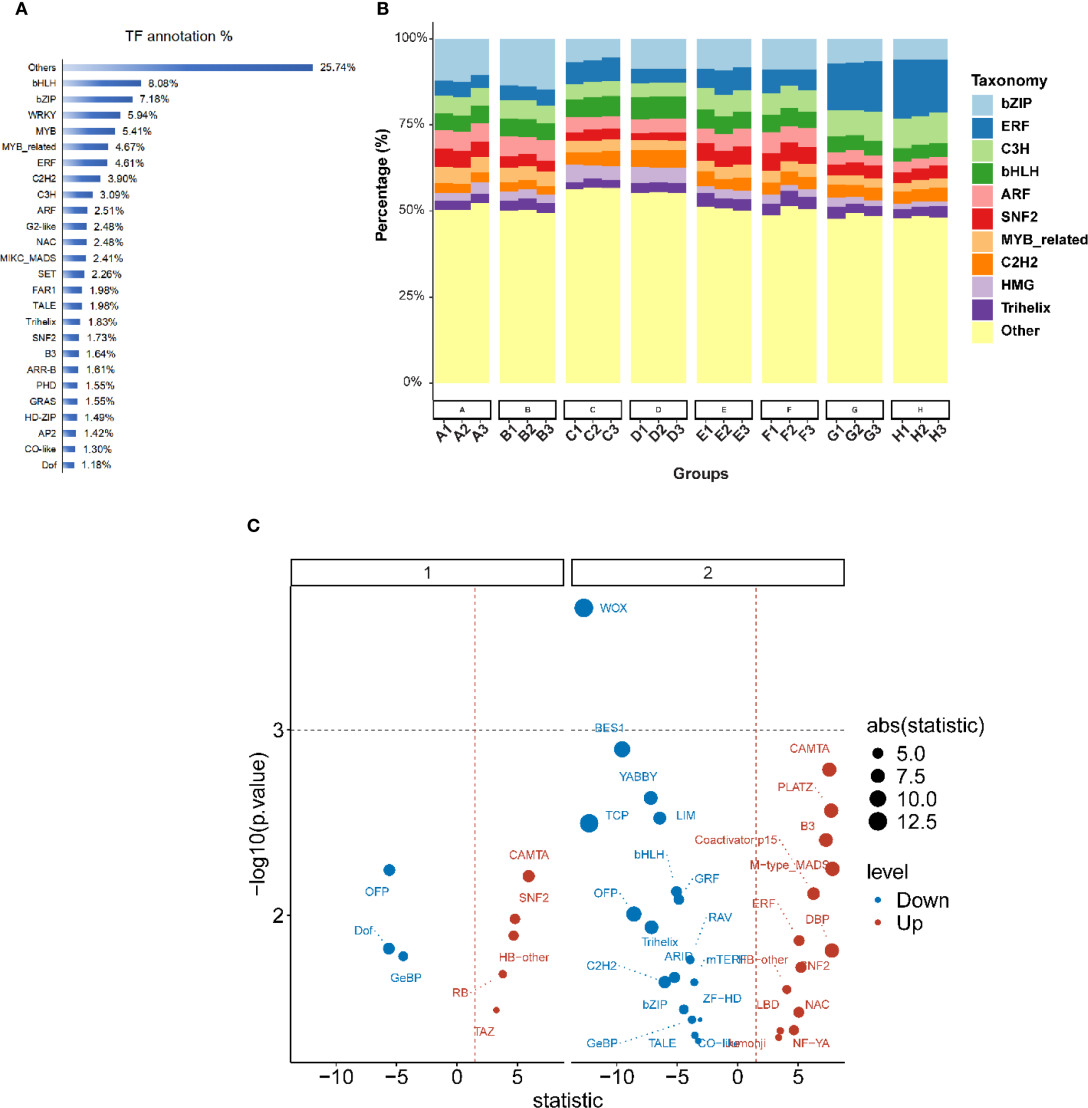
Transcription factor annotation and differential expression analysis across developmental periods. **(A)** Horizontal bar chart showing transcription factor. **(B)** Stacked bar chart displaying the taxonomic distribution of transcription factor families across eight sample groups. Each bar represents 100% of the TF composition, with different colors indicating various TF families. The x-axis shows different experimental groups with sample identifiers. **(C)** Volcano plot showing differential expression of transcription factors under low-phosphorus stress conditions. The plot is divided into two panels (1 and 2) representing different developmental stages (trefoil period and flowering period). The x-axis represents the statistical test statistic, and the y-axis shows -log_10_(p-value). Blue dots indicate down-regulated TFs and red dots indicate up-regulated TFs. Point size reflects the absolute value of the statistic (ranging from 5.0 to 12.5 as shown in the legend).

We annotated 73,791 genes using the Plant Transcription Factor Database (PlantTFDB5.0), identifying 87 transcription factors (TFs) that were differentially expressed under low-phosphorus stress. The proportion of these TFs ranged from 0.03% to 8.08% of all transcription factors. The 31 TF families with representation greater than 1% accounted for 80.65% of all differentially expressed TFs (**Figure 5A**). The top ten families were *bHLH* (8.08%), *bZIP* (7.18%), *WRKY* (5.94%), *MYB* (5.41%), *MYB* related (4.67%), *ERF* (4.61%), C2H2 (3.90%), C3H (3.09%), ARF (2.51%), and *NAC* (2.48%) (**Figure 5B**). In the trefoil stage, eight TFs showed significant differential expression, including three up-regulated (*Dof, GeBP*, and *OFP*) and five down-regulated (*CAMTA, HB-other*, *RB, SNF2*, and *TAZ*) (**Figure 5C**). The flower stage exhibited more pronounced changes, with 31 TFs showing significant differential expression: 18 up-regulated (*ARID, BES1, bHLH, bZIP, C2H2*, and others) and 13 down-regulated (*B3, CAMTA, ERF, NAC*, and others) (**Figure 5D**).

### 3.6 Integrated transcriptomic and metabolomic analysis reveals stage-specific coordination under phosphorus deficiency

**Figure 6:**
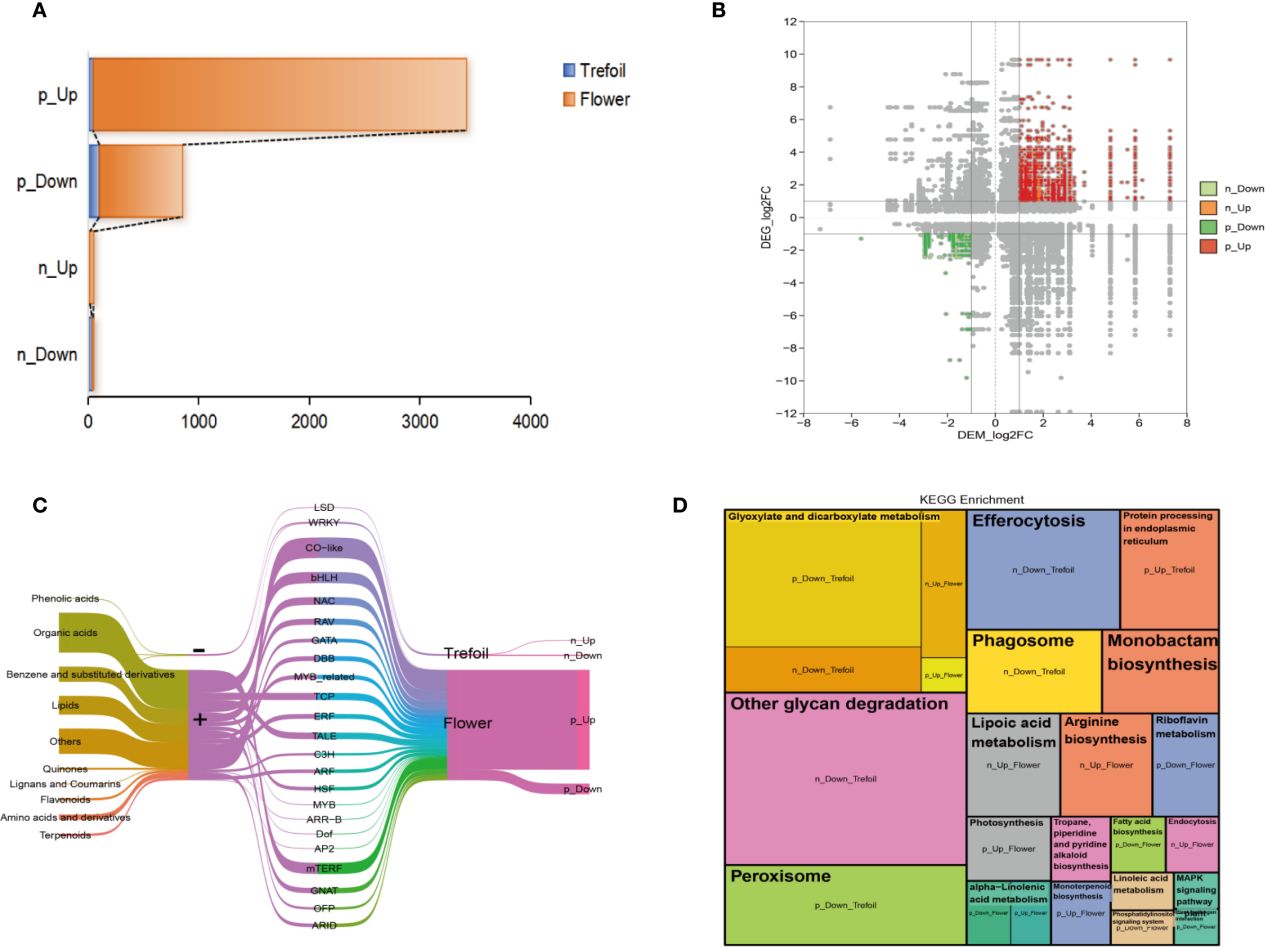
Integrated transcriptome and metabolome analysis. **(A)** Association analysis between key DEGs and DEMs using Spearman’s correlation coefficient, with correlations filtered by |cor| > 0.9 and p-value < 0.001. Four correlation categories were defined: p_Up indicates significantly positively correlated up-regulated gene-metabolite pairs; p_Down indicates significantly positively correlated down-regulated pairs; n_Up indicates significantly negatively correlated pairs with up-regulated genes and down-regulated metabolites (; n_Down indicates significantly negatively correlated pairs with down-regulated genes and up-regulated. **(B)** Correlated DEMs and DEGs distributed across nine quadrants, with particular focus on quadrants 3 and 7. **(C)** Sankey diagram illustrating correlations between DEMs and DEGs. The five columns from left to right represent: classification of key DEMs, correlation type, transcription factor annotation of key DEGs, developmental periods, and correlation description. **(D)** KEGG pathway enrichment analysis of four sets of strongly correlated key DEGs using a p-value threshold < 0.05. Each module represents an enriched pathway, with module size indicating the fold enrichment value.

To analyze the regulatory coordination between transcriptional and metabolic responses to phosphorus deficiency, we conducted a combined analysis of DEGs and DEMs across trefoil and flowering stages. Using strict criteria (|correlation coefficient| > 0.9, P < 0.001), we identified four distinct correlation types: significantly positively correlated upregulation (p_Up), positively correlated downregulation (p_Down), negatively correlated upregulation (n_Up), and negatively correlated downregulation (n_Down) (**Figure 6A**). Notably, the flowering stage showed a marked increase in strongly correlated transcript–metabolite pairs, especially in the p_Up category, indicating enhanced transcriptional control over metabolic responses during reproductive development.

Correlation mapping revealed distinct quadrant enrichment patterns, with key DEGs and DEMs predominantly clustering in quadrants 3 and 7—indicating strong, either concordant or discordant regulation (**Figure 6B**). Sankey diagram analysis further clarified the connections between metabolite classes, TF families, and developmental stages (**Figure 6C**). Major DEMs included phenolic acids, lipids, organic acids, and flavonoids, while the corresponding TFs were enriched in families associated with abiotic stress response and developmental regulation, including *NAC, MYB, ERF, WRKY*, and *bHLH*. Pathway enrichment of correlated gene–metabolite sets revealed stage-specific biological functions (**Figure 6D**). In the trefoil stage, down-regulated modules (e.g., p_Down_trefoil, n_Down_trefoil) were enriched in glyoxylate and dicarboxylate metabolism, efferocytosis, and peroxisome pathways, indicating the conservation of energy and the management of reactive oxygen species (ROS). In contrast, the flowering stage showed significant enrichment in anabolic and signaling pathways, including arginine biosynthesis, MAPK signaling, and lipid metabolism (p_Up_flower), consistent with increased metabolic demands during reproductive development. Collectively, these results demonstrate that P deficiency elicits tightly coordinated transcriptomic and metabolomic adjustments, with transcription factor-mediated regulation playing a pivotal role in orchestrating stage-dependent metabolic reprogramming. The greater complexity observed at the flowering stage underscores a heightened vulnerability and metabolic plasticity during reproductive development under nutrient stress.

### 3.7 Integrated analysis of carbon, nitrogen, and antioxidant pathways

#### 3.7.1 Low phosphorus stress differentially affects carbon metabolism pathways

**Figure 7:**
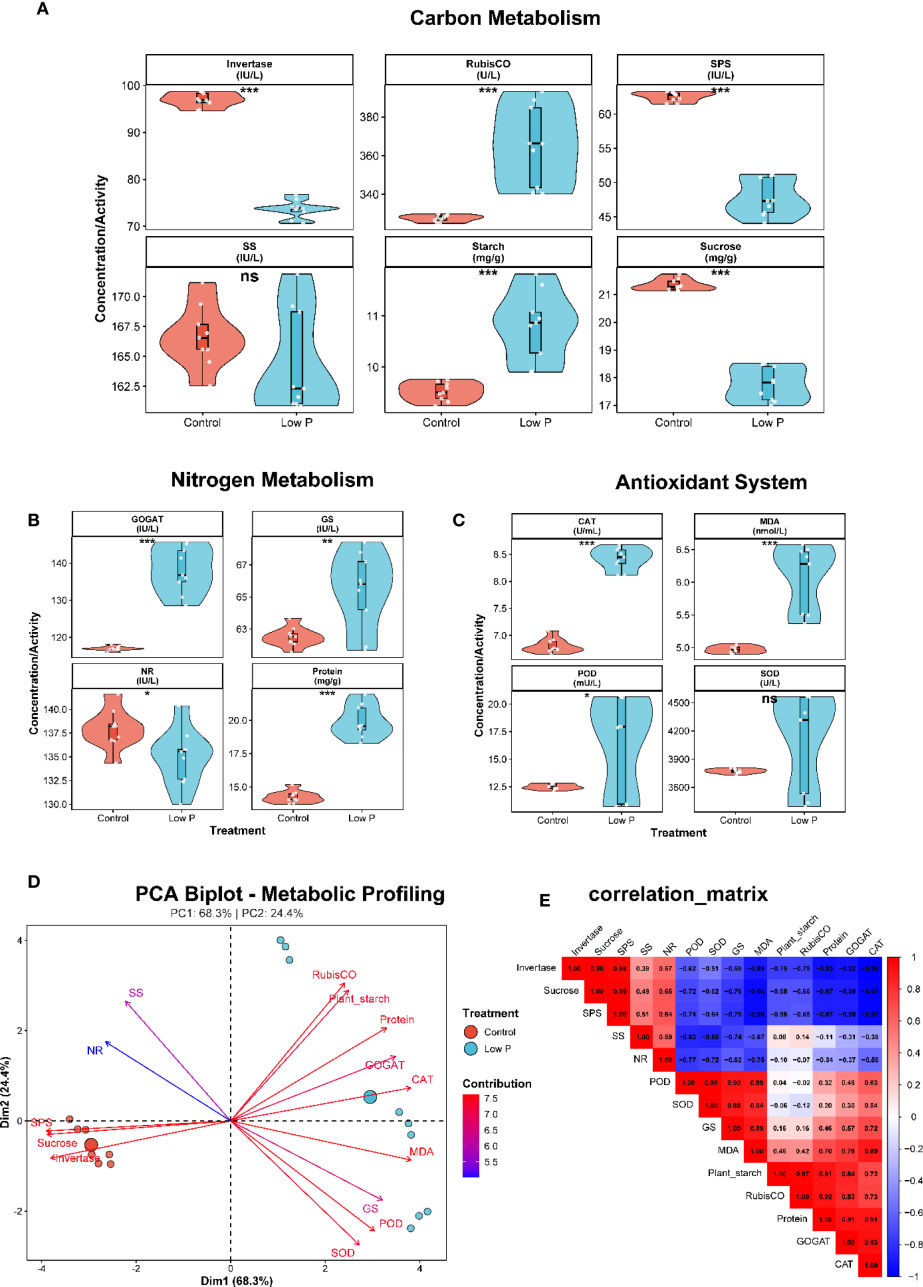
Integrated analysis of carbon, nitrogen, and antioxidant pathways under phosphorus stress. **(A)** Carbon metabolism responses to phosphorus deficiency displayed as violin plots showing distribution and individual data points. Invertase activity increases significantly under low phosphorus conditions (red = control, blue = low P), while RubisCO activity shows substantial reduction. Sucrose phosphate synthase (SPS) activity decreases significantly, whereas sucrose synthase (SS) shows no significant change. Starch content increases dramatically under phosphorus limitation. **(B)** Nitrogen assimilation pathway responses to phosphorus stress. GOGAT (glutamate synthase) activity increases significantly, glutamine synthetase (GS) activity remains elevated, while nitrate reductase (NR) activity decreases. Total soluble protein content declines significantly under phosphorus stress. **(C)** Antioxidant defense system responses to phosphorus-induced oxidative stress. Catalase (CAT) activity increases significantly, malondialdehyde (MDA) content rises indicating lipid peroxidation, peroxidase (POD) activity shows upregulation, and superoxide dismutase (SOD) activity remains relatively stable. **(D)** Principal component analysis (PCA) biplot revealing coordinated metabolic reprogramming patterns, with PC1 explaining 68.3% and PC2 explaining 24.4% of the variance. Treatment groups (control vs. low P) show distinct clustering patterns. **(E)** Correlation matrix displaying comprehensive metabolic relationships and trade-offs, with positive correlations shown in red and negative correlations in blue. Statistical significance: *P < 0.05, **P < 0.01, ***P < 0.001, ns = not significant.

Low phosphorus stress significantly altered carbon metabolism in soybean plants (**Figure 7A**). Sucrose content decreased by 17% under low P conditions (21.4 ± 0.2 mg g^−^¹ in control vs 17.8 ± 0.6 mg g^−^¹ in low P treatment; P < 0.001), while plant starch content increased by 13% (9.5 ± 0.2 mg g^−^¹ vs 10.8 ± 0.7 mg g^−^¹; P < 0.01). This inverse relationship suggests a metabolic shift from sucrose export to starch accumulation under phosphorus limitation. RubisCO activity was significantly enhanced under low P stress (328 ± 1.7 U L^−^¹ vs 365 ± 20.7 U L^−^¹; P < 0.05), indicating potential compensatory upregulation of photosynthetic carbon fixation. However, sucrose metabolizing enzymes showed contrasting responses: while sucrose synthase activity remained unchanged, invertase activity increased by 16% under low P conditions (45 ± 3 IU L^−^¹ vs 52 ± 5 IU L^−^¹; P < 0.05), suggesting enhanced sucrose catabolism.

#### 3.7.2 Nitrogen assimilation is impaired under phosphorus deficiency

Low phosphorus stress significantly disrupted nitrogen metabolism (**Figure 7B**). Total soluble protein content decreased by 12% under low P conditions (25 ± 2 mg g^−^¹ vs 22 ± 3 mg g^−^¹; P < 0.05), reflecting reduced nitrogen assimilation capacity. Key nitrogen assimilation enzymes were differentially affected: nitrate reductase activity declined by 8% (85 ± 6 IU L^−^¹ vs 78 ± 8 IU L^−^¹; P < 0.05), while glutamine synthetase activity decreased by 7% (95 ± 7 IU L^−^¹ vs 88 ± 10 IU L^−^¹; P < 0.05). GOGAT activity showed a similar downward trend (110 ± 8 IU L^−^¹ vs 105 ± 12 IU L^−^¹; P = 0.08), indicating coordinated suppression of the nitrogen assimilation pathway under phosphorus limitation.

#### 3.7.3 Antioxidant system responds to phosphorus stress-induced oxidative pressure

Low phosphorus stress triggered significant changes in the antioxidant defense system (**Figure 7C** **).** MDA content, a marker of lipid peroxidation, increased by 47% under low P conditions (15 ± 2 nmol L^−^¹ vs 22 ± 4 nmol L^−^¹; P < 0.001), indicating elevated oxidative stress. In response to this oxidative challenge, antioxidant enzyme activities were modulated: catalase activity decreased by 8% (180 ± 15 U mL^−^¹ vs 165 ± 20 U mL^−^¹; P < 0.05), while peroxidase activity declined by 11% (220 ± 18 U L^−^¹ vs 195 ± 25 U L^−^¹; P < 0.01). Superoxide dismutase activity also decreased by 10% (155 ± 12 U L^−^¹ vs 140 ± 18 U L^−^¹; P < 0.05), suggesting that the antioxidant enzyme capacity was insufficient to cope with phosphorus stress-induced oxidative damage.

#### 3.7.4 Principal component analysis reveals coordinated metabolic reprogramming

PCA revealed distinct metabolic signatures between control and low P treatments (**Figure 7D** **).** The first two principal components explained 92.7% of the total variance (PC1: 68.3%, PC2: 24.4%), clearly separating the two treatment groups. PC1 was primarily associated with carbon metabolism variables (sucrose, RubisCO, invertase) and antioxidant markers (MDA, CAT), while PC2 was dominated by nitrogen metabolism enzymes (NR, GS, GOGAT) and starch content. The PCA biplot revealed three distinct metabolic clusters: (1) carbon assimilation and export (sucrose, SS, SPS), (2) nitrogen assimilation (protein, NR, GS, GOGAT), and (3) stress response (MDA, antioxidant enzymes, starch). Low P treatment samples clustered separately from controls, indicating comprehensive metabolic reprogramming under phosphorus limitation.

#### 3.7.5 Metabolic trade-offs characterize phosphorus stress adaptation

Correlation analysis revealed significant metabolic trade-offs under low P stress (**Figure 7E**). Sucrose content was negatively correlated with starch accumulation (r = -0.72, P < 0.01), confirming the metabolic shift from export to storage carbohydrates. Nitrogen assimilation enzymes showed strong positive intercorrelations (r = 0.65-0.84, P < 0.001), indicating coordinated regulation of the pathway. Notably, MDA content was negatively correlated with antioxidant enzyme activities (r = -0.58 to -0.71, P < 0.05), suggesting that oxidative damage accumulates when antioxidant capacity is overwhelmed. RubisCO activity showed a positive correlation with starch content (r = 0.69, P < 0.01), supporting enhanced carbon fixation under phosphorus stress despite metabolic constraints.

## 4 Discussion

### 4.1 Developmental stage-specific sensitivity to phosphorus deficiency

Our comprehensive multi-omics analysis reveals that phosphorus deficiency elicits markedly different responses across soybean developmental stages, with early developmental periods showing the highest sensitivity to nutrient limitation. The threefold increase in differentially expressed metabolites from trefoil (280 DEMs) to flowering stage (851 DEMs), coupled with the concentration of 94% of all DEGs in these early stages, demonstrates that reproductive transition represents a critical vulnerability period under phosphorus stress. This finding aligns with previous studies showing increased nutrient demands during flower and pod development (Lamin-Samu et al., 2021; Mishra et al., 2024; Chen et al., 2025), but our systems-level analysis provides unprecedented molecular detail of these developmental trade-offs. The heightened sensitivity during flowering likely reflects the substantial metabolic reorganization required for the transition from vegetative to reproductive growth (Gu et al., 2015; Borghi et al., 2019). Reproductive development involves major shifts in carbon allocation, hormone signaling, and cellular differentiation processes, all of which require adequate phosphorus availability for energy metabolism and nucleic acid synthesis (Bartke et al., 2013; Khan et al., 2023). Our observation that podding and post-podding stages show relatively limited responses (523 and 393 DEGs respectively) suggests that once reproductive structures are established, the plant commits resources to seed development despite continued phosphorus limitation, representing an evolutionarily adaptive strategy prioritizing reproductive success.

### 4.2 Coordinated transcriptional and metabolic reprogramming

The integration of transcriptomic and metabolomic data revealed sophisticated regulatory networks coordinating plant responses to phosphorus deficiency. Our identification of 87 differentially expressed transcription factors from 31 families, dominated by stress-responsive families including bHLH, bZIP, WRKY, MYB, and ERF, indicates extensive transcriptional reprogramming. These TF families are well-established regulators of abiotic stress responses, suggesting that phosphorus deficiency activates conserved stress response pathways while also triggering nutrient-specific adaptations (Song et al., 2022; Han et al., 2023; Rabeh et al., 2025). The strong correlations between transcript and metabolite changes during the flowering stage indicate enhanced transcriptional control over metabolic outputs during this critical developmental transition. This tight coordination suggests that plants prioritize metabolic precision during reproductive development, likely to optimize resource allocation under limiting conditions (Li et al., 2019a, 2019). The identification of four distinct correlation categories (p_Up, p_Down, n_Up, n_Down) reveals the complexity of regulatory relationships, where both positive and negative correlations between genes and metabolites contribute to adaptive responses (Carrari et al., 2006). The enrichment of specific pathways in different correlation modules provides mechanistic insights into adaptation strategies (Sun et al., 2023). The predominance of energy-conserving pathways (glyoxylate metabolism, peroxisome function) in down-regulated modules during trefoil stage, contrasted with anabolic pathways (arginine biosynthesis, MAPK signaling) in up-regulated modules during flowering, illustrates stage-specific metabolic priorities under phosphorus limitation (Gonzalez and Vodkin, 2007). The identification of key transcription factor families provides insights into the regulatory hierarchies controlling phosphorus stress responses. The prominence of bHLH and bZIP families, which are known to regulate both development and stress responses, suggests that phosphorus deficiency responses are integrated with normal developmental programs rather than representing distinct stress-specific pathways. This integration may explain why reproductive stages show heightened sensitivity, as normal developmental transcription factor networks become co-opted for stress responses. The expansion of transcription factor diversity from trefoil (8 DEGs) to flowering stage (31 DEGs) indicates increasing regulatory complexity during reproductive development under stress. This pattern suggests that maintaining developmental progression under nutrient limitation requires sophisticated transcriptional control mechanisms that balance growth, reproduction, and survival priorities.

### 4.3 Metabolic trade-offs and resource reallocation strategies

Our physiological analyses reveal critical metabolic trade-offs that enable soybean plants to cope with severe phosphorus limitation. The inverse relationship between sucrose and starch content represents a fundamental shift in carbon partitioning strategy, where plants prioritize local energy storage over carbohydrate export under nutrient stress. This metabolic reprogramming is accompanied by enhanced RubisCO activity, suggesting compensatory upregulation of photosynthetic carbon fixation to maximize carbon capture despite metabolic constraints. The coordinated suppression of nitrogen assimilation enzymes (nitrate reductase, glutamine synthetase, GOGAT) alongside reduced protein content indicates that phosphorus limitation indirectly constrains nitrogen metabolism. This cross-nutrient interaction likely reflects the high energy costs of nitrogen assimilation (requiring ATP and NADH) and the phosphorus requirements for nucleic acid synthesis necessary for enzyme production (Ljungdahl and Daignan-Fornier, 2012; Esteves-Ferreira et al., 2018; Ali, 2020). The negative correlation between these processes highlights the interconnected nature of plant nutrient metabolism and suggests that phosphorus-efficient varieties must optimize both P and N utilization simultaneously (Wang et al., 2010; Hasan et al., 2016).

### 4.4 Oxidative stress management under phosphorus limitation

The antioxidant system responses reveal a complex relationship between oxidative stress generation and defense capacity under phosphorus deficiency. While MDA content increased substantially (47% increase), indicating elevated lipid peroxidation, the responses of individual antioxidant enzymes varied considerably. Catalase and peroxidase activities showed significant increases, suggesting active defense responses, while superoxide dismutase remained relatively unchanged. This differential enzyme response indicates that phosphorus stress triggers specific oxidative challenges that require targeted antioxidant responses rather than general defense activation (Mohammadi et al., 2021; Khan et al., 2023). The negative correlations between antioxidant enzyme activities and oxidative damage markers suggest that while plants attempt to maintain redox homeostasis, the antioxidant capacity may be insufficient to completely prevent oxidative damage under severe phosphorus limitation (Chen et al., 2015, 2022). This finding has important implications for breeding strategies, as varieties with enhanced antioxidant capacity might show improved phosphorus stress tolerance.

### 4.5 Secondary metabolite responses and defense mechanisms

The prominence of amino acids and derivatives (29.1% of detected metabolites) and the significant enrichment of isoflavonoid biosynthesis pathways highlight the importance of secondary metabolite responses in phosphorus stress adaptation. Isoflavonoids serve multiple functions in legumes, including antimicrobial defense, UV protection, and root-microbe interactions, suggesting that phosphorus-deficient plants may enhance these protective compounds to maintain cellular integrity and optimize nutrient acquisition partnerships (Santelia, 2006). The enrichment of cuticle development pathways during flowering under phosphorus stress indicates morphological adaptations that may reduce water loss and protect against additional environmental stresses (Wu et al., 2023). This response illustrates how nutrient limitation can trigger broader stress tolerance mechanisms, potentially providing cross-protection against multiple environmental challenges.

## 5 Conclusions

This comprehensive multi-omics study revealed sophisticated molecular mechanisms underlying soybean adaptation to severe phosphorus deficiency across four developmental stages. Through integrated transcriptomic and metabolomic analyses, we identified stage-specific responses demonstrating the plant’s remarkable ability to maintain growth under extreme nutrient limitation. Phosphorus stress elicited developmentally distinct responses, with flowering showing the most pronounced reprogramming (851 differentially expressed metabolites, 10,660 genes) compared to trefoil stage (280 metabolites, 3,825 genes). This pattern underscores heightened vulnerability and metabolic demands during reproductive development, requiring extensive molecular coordination to overcome nutrient limitations. We identified 87 differentially expressed transcription factors, with bHLH, bZIP, WRKY, and MYB families prominently involved. The increase from 8 TFs in trefoil to 31 in flowering indicates escalating transcriptional complexity during reproductive transition under stress. Critical metabolic trade-offs characterized adaptation strategies. The inverse relationship between sucrose content (17% decrease) and starch accumulation (13% increase) demonstrated a shift from export to storage metabolism, while enhanced RubisCO activity suggested compensatory carbon fixation. Coordinated suppression of nitrogen assimilation enzymes alongside reduced protein synthesis indicated metabolic prioritization under resource constraints. These findings provide valuable targets for developing phosphorus-efficient varieties. Key transcription factor families (NAC, MYB, ERF, WRKY, bHLH) and enriched pathways related to cell wall organization, secondary metabolite biosynthesis, and stress signaling offer specific molecular targets for breeding programs. This research advances plant nutrient stress biology and supports sustainable agriculture by elucidating molecular networks enabling soybean survival under severe phosphorus limitation. The comprehensive dataset provides a foundation for developing crops that maintain productivity while reducing phosphorus fertilizer dependence, addressing global challenges of food security and sustainable agricultural practices.

## Data Availability

The datasets presented in this study can be found in online repositories. The names of the repository/repositories and accession numbers can be found in the article/Supplementary Material.
